# Polyendocrine metabolic ovarian syndrome (PMOS)/polycystic ovary syndrome (PCOS): current and future trends

**DOI:** 10.1172/JCI202824

**Published:** 2026-06-15

**Authors:** Jessica L. Chan, Irene Masini, Margareta D. Pisarska

**Affiliations:** Department of Obstetrics and Gynecology, Cedars-Sinai Medical Center, Los Angeles, California, USA.

## Abstract

Polycystic ovary syndrome (PCOS), also known as polyendocrine metabolic ovarian syndrome (PMOS), is the most common endocrinologic disorder to affect women. Despite this, the pathophysiology of the disease is not entirely known. This has hindered the diagnosis of the disease and appropriate treatment for millions of individuals. In this Review, we discuss the proposed pathophysiology of PCOS from a translational perspective. We review the existing diagnostic criteria of PCOS and current management strategies. Finally, we discuss the long-term health sequelae associated with PCOS, future directions, and areas of needed research in this often-overlooked disease.

## Introduction

Polycystic ovary syndrome (PCOS) is the most common endocrinopathy in women, affecting 5%–20% of the population, depending on the criteria used (1). A combination of increased androgens and/or insulin underlies the hormonal imbalance characteristic of this disorder. Individuals with PCOS are at an elevated risk for several medical comorbidities, including menstrual cycle irregularities, infertility, and metabolic dysfunction, including insulin resistance, type 2 diabetes mellitus, dyslipidemia, hypertension, and cardiovascular disease (CVD) (1). Mental health disorders, including depression and anxiety, as well as disordered eating and poor body image, are also common among individuals with PCOS, leading to greatly worsened quality of life (2). Despite concerted efforts to determine a single set of criteria for the diagnosis of PCOS, a global agreement has not been reached. Genetic and environmental contributions are thought to influence disease presentation and may contribute to differences seen in PCOS phenotypes. Here, we review diagnostic criteria of PCOS, the proposed pathophysiology, management strategies, long-term health sequelae, and future research directions.

## Diagnosis of PCOS

The 2023 international evidence-based guidelines for the assessment and management of PCOS recommend using the Rotterdam criteria for the diagnosis in adults, with a requirement of two out of the following three characteristics: (a) ovulatory dysfunction (OD), (b) clinical or biochemical hyperandrogenism (HA), and (c) polycystic ovary morphology (PCOM) on ultrasound, with exclusion of other hormonal disorders (Table 1) (3, 4). Given that pathological ovarian cysts are not a requirement for diagnosis, an international consortium recently renamed the condition polyendocrine metabolic ovarian syndrome (PMOS) (5). 

OD often presents as irregular menstrual cycles, defined as a cycle of <21 or >35 days, from 3 years after menarche until perimenopause. HA can be clinical or biochemical. Clinical HA often presents as hirsutism, acne, or alopecia. The modified Ferriman-Gallwey score is used as an objective assessment of hirsutism. A score of 4 to 6 can be used to detect hirsutism. However, this can be variable based on ethnicity and self-treatment. A score of ≥8 is used to define hirsutism in Black or White women in the United States and United Kingdom, but a score of ≥2 has been used in Asian women (3, 6). This scoring system is limited because of its subjective nature. Therefore, the term “patient-important hirsutism” has been suggested to describe unwanted sexual hair growth that causes enough distress to seek treatment (6). Biochemical HA is determined through measurement of total and free testosterone. Total testosterone should be measured with highly sensitive tandem mass spectrometry (LC-MS/MS). Free testosterone should be measured by calculation, equilibrium dialysis, or ammonium sulfate precipitation. If not available, free testosterone can be estimated by the calculated free androgen index. If total or free testosterone levels are not elevated, clinicians may consider measuring androstenedione and DHEAS. These measures are less specific for PCOS/PMOS, and age-related cutoffs should be applied. PCOM is ideally evaluated via pelvic transvaginal ultrasonography with transducer bandwidth of ≥8 MHz, is defined as ≥20 antral follicles between 2 and 9 mm in at least one ovary or a volume ≥10 mL, and can assist with PCOS/PMOS diagnosis in individuals who present with only one other Rotterdam criterion (7, 8). Special training and standardized protocols are needed for PCOM reporting. Due to this need for standardized assessment, the most recent international guidelines state that AMH level may be used as an alternative, checked at least 8 years postmenarche because of the strong correlation between antral follicle count and AMH. AMH alone should not be used as a singular diagnostic test for PCOS/PMOS. While “high” levels of AMH have been associated with PCOS/PMOS, no specified values have been determined to define PCOM (3). A study using a fully automated Elecsys AMH assay found that, in women between 23 and 35 years of age, an AMH above 3.2 ng/mL as a surrogate for PCOM had a sensitivity of 88.6% and a specificity of 80.3% (9).

Others have suggested defining PCOS/PMOS by phenotype (10). The different combinations of diagnostic criteria lead to four possible phenotypes: A) HA+OD+PCOM, B) HA+OD, C) HA+PCOM, and D) OD+PCOM. Each phenotype is associated with its own unique metabolic risk, with phenotype A having the highest rates of insulin resistance, metabolic syndrome, and dyslipidemia and phenotype D having the mildest metabolic profile (10).

There are two other diagnostic criteria that are used, to some extent. The National Institutes of Health criteria require HA and OD, and the Androgen Excess PCOS Society criteria require the presence of HA and either OD or PCOM (11, 12).

The varying diagnostic criteria make defining PCOS/PMOS and determining best treatment practice challenging and complicate epidemiologic studies to determine the true prevalence of PCOS/PMOS. However, simple screening tools have high predictive value to identify individuals at risk for androgen excess disorders, including PCOS/PMOS (13). For example, in one study, participants were asked three questions to self-assess the presence/absence of male-like hair and menstrual irregularity. The calculated sensitivity and specificity of this questionnaire to predict PCOS/PMOS were 89% and 78%, respectively (13).

## Pathophysiology of PCOS/PMOS

**Figure 1:**
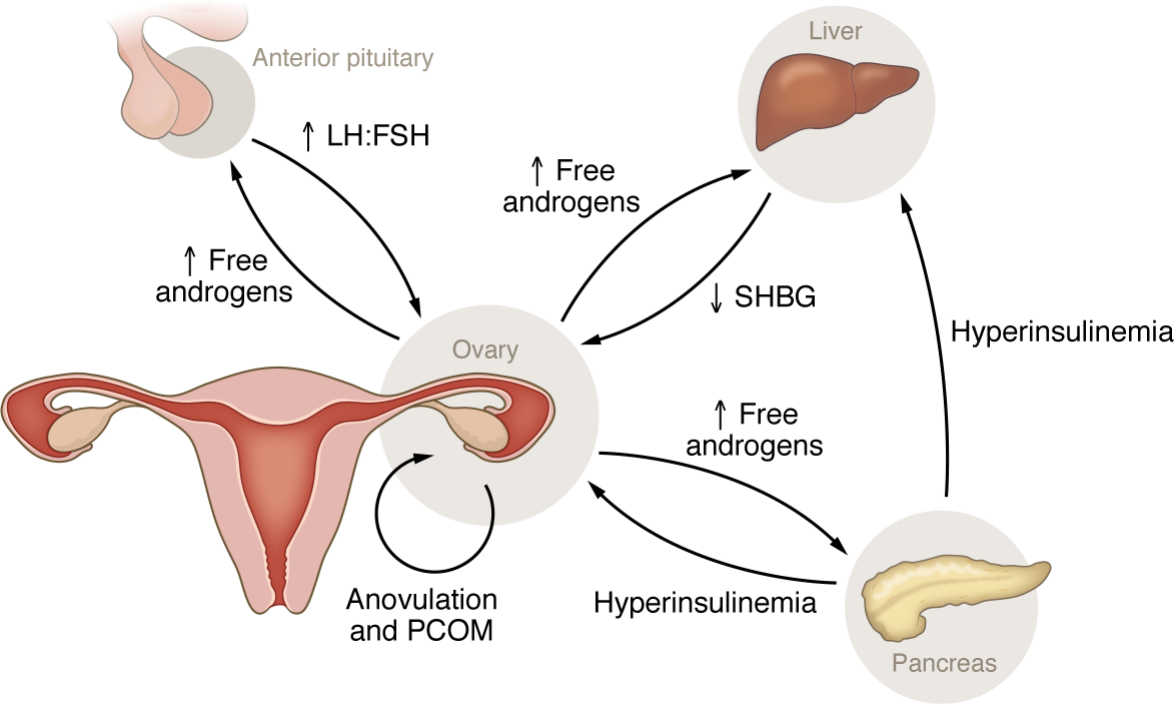
PCOS/PMOS pathophysiology. PCOS/PMOS is a complicated syndrome, involving multiple feedback loops in the anterior pituitary, liver, pancreas, and ovary ultimately contributing to a hyperandrogenic state. In the pituitary, LH is released in higher quantities than FSH, simulating the theca cells of the ovary to increase production of androgens. The androgens in turn stimulate the pituitary to release LH and FSH. The increased androgen also acts on the liver, decreasing SHBG, which increases circulating free androgens. Finally, at the level of the pancreas, androgens lead to hyperinsulinemia, which in turn also decreases SHBG. Insulin also acts on the theca cells to promote androgen release. All of these factors lead to HA, which leads to anovulation and PCOM in the ovary. LH, luteinizing hormone; FSH, follicle-stimulating hormone; SHBG, sex hormone–binding globulin.

The pathophysiology of PCOS/PMOS is complex and multifactorial, making it difficult for a single theory to account for its development (Figure 1). There are genetic, epigenetic, and environmental factors that modulate neuroendocrine, ovarian, and metabolic functions, and it is the interplay among these components that ultimately leads to PCOS/PMOS development (14). Further understanding these mechanisms has uncovered new targets in advancements of its diagnosis and treatment.

### Role of genetics and epigenetics.

PCOS/PMOS is highly heritable, with 60%–70% of daughters born to mothers with PCOS/PMOS ultimately diagnosed with PCOS/PMOS as well (15). Genome-wide association studies (GWAS) have implicated different genes (16–21) and proteins (22) in the development of PCOS/PMOS (Table 2). Two large-scale GWAS on individuals of Han Chinese ancestry identified 11 genes that were strongly associated with PCOS/PMOS: *LHCGR*, *FSHR*, *THADA*, *C9orf3*, *DENND1A*, *YAP1*, *RAB5B/SUOX/ERBB3*, *HMGA2*, *OX3*, *INSR*, and *SUMO1P1* (16, 17). Three other large-scale GWAS on individuals of European ancestry also found an association between PCOS/PMOS and many of these same genes, in addition to *ERRB4*, *IRF1/RAD50*, *GATA4/NEIL2*, *PLGRKT*, *FSHB*, *ZBTB16*, *KRR1*, and *MAPRE* (18–20). The majority of these genes encode proteins involved in insulin signaling, sex hormone function, and regulation of metabolism, processes associated with PCOS/PMOS (23). These gene loci are conserved among the different PCOS/PMOS phenotypes, suggesting a similar overall genetic architecture among patients with varying presentation (23). However, a recent analysis using distinct clinical subtypes of PCOS/PMOS determined by clustering of reproductive, metabolic, and background traits demonstrated unique genetic profiles in each group (24). Notably, variants in the genes listed above accounted for less than 10% of PCOS/PMOS prevalence (25), which may in part be due to the fact that GWAS primarily identify common variants associated with small effect sizes (26). Furthermore, GWAS identify statistical associations and not the mechanisms by which these genes contribute to PCOS/PMOS pathogenesis. The missing heritability also highlights the likely contribution of additional factors to the pathophysiology of PCOS/PMOS.

Epigenetics describes the study of gene expression heritability that occurs without changes to the DNA sequence itself (27). These changes may occur due to exposure to certain chemicals that can mimic endogenous hormones, both in utero and during important development periods. For example, exposure to valproate, the antiepileptic medication, has been associated with development of the PCOS/PMOS phenotype (28, 29). There is also emerging evidence that environmental toxins, including endocrine-disrupting chemicals from everyday items, can interfere with hormone signaling and metabolic pathways via epigenetic mechanisms, promoting features of PCOS/PMOS (30, 31). Differences in presentation of PCOS/PMOS in two states of the United States, Alabama and California, suggest a potential role of the environment on PCOS/PMOS, though further research needs to be done to identify whether epigenetics plays a role (32). DNA methylation, a common epigenetic mechanism that controls gene expression, has been implicated in the development of PCOS/PMOS in several studies (25, 33). A meta-analysis found the DNA of individuals with PCOS/PMOS to be globally hypomethylated in various tissues and peripheral blood but hypermethylated at genes including *INSR*, which encodes the insulin receptor, and specifically hypomethylated at other genes such as *AMH* and *AMHR*, which encode AMH and its receptor (34). Several studies, primarily on animal models, have linked exposure to high levels of androgens in utero to the development of PCOS/PMOS in offspring via epigenetic mechanisms, including changes in DNA methylation (28, 35, 36). Other studies in animal models have shown that reversing hypomethylation using methyl donors can restore normal expression of those genes and restore normal reproduction and metabolic function (37, 38), offering a possible target for future treatment.

### Roles of HA and insulin resistance.

HA is present in greater than 80% of individuals with PCOS/PMOS (25). The main driver is androgen production within ovarian theca cells, though adrenal androgen production may also play a role (39). Production of androgens within theca cells is mainly stimulated by LH (40). In addition, insulin resistance, which is present in up to 70% of individuals with PCOS/PMOS, contributes to androgen excess in several ways (41). First, hyperinsulinemia decreases SHBG, increasing the levels of circulating androgens (25). Furthermore, insulin has gonadotropic properties on ovarian tissue, driving androgen production (41). Hyperinsulinemia leads to increased sensitization of theca cells to LH, further promoting theca cell production of androgens (42). In a mouse model of PCOS/PMOS, knocking out the insulin receptor gene on theca cells corrected HA (43). Hyperinsulinemia has also been shown to inhibit the maturation of follicles, leading to anovulation and PCOM (25, 41). Insulin resistance also correlates with the degree of anovulation seen in patients with PCOS/PMOS (44).

Moreover, obesity, which is highly prevalent among patients with PCOS/PMOS, is associated with pancreatic β cell dysfunction and impaired insulin signaling (42, 45). It is hypothesized that the clinical heterogeneity of PCOS/PMOS may be explained by the interplay between androgen excess and metabolic triggers including obesity and hyperinsulinemia, with some individuals developing PCOS/PMOS due to an intrinsic androgen abnormality alone, while those with a milder defect may require additional metabolic stressors to manifest the condition (28, 46, 47). Importantly, not all individuals with insulin resistance have PCOS/PMOS, and not all individuals with PCOS/PMOS are insulin resistant, indicating additional factors at play (28).

### Roles of inflammation and neuroendocrine signaling.

There is evidence that low-grade chronic inflammation plays a role in the long-term metabolic consequences of PCOS/PMOS (48). Individuals with PCOS/PMOS exhibit higher levels of inflammatory markers, such as C-reactive protein; however, chronic inflammation is also common in individuals with obesity or insulin resistance, so it is difficult to determine whether it is a causal driver of PCOS/PMOS (49). Chronic inflammation may arise from the relatively lower levels of progesterone due to anovulation, as progesterone exhibits antiinflammatory properties (49). Additionally, altered fatty acid metabolism in the follicular fluid from individuals with PCOS/PMOS leads to inflammasome activation via the ERK1/2 signaling pathway (50). Alternatively, recent data demonstrate that immune suppression and impaired angiogenic signaling may play a role in PCOS/PMOS, rather than chronic inflammation (51), complicating our understanding of the role of inflammatory cytokines in PCOS/PMOS pathogenesis.

There is also increasing evidence that the etiology of androgen excess in PCOS/PMOS involves abnormal gonadotropin-releasing hormone (GnRH) signaling in the hypothalamus (52). GABA, which is usually an inhibitory neurotransmitter, has been shown to increase activity of GnRH neurons (53). Previous studies demonstrated an increase in GABA innervation of GnRH neurons in individuals with PCOS/PMOS (54), and more recent data demonstrate an increase in GABA signaling to GnRH neurons in mice with prenatal androgen exposure (55), supporting the likely important role of GABA in the development of PCOS/PMOS. Neurons located in the arcuate nucleus of the hypothalamus releasing kisspeptin, neurokinin B, and dynorphin play a pivotal role in the pulse generation of GnRH neurons and have been implicated in the abnormal signaling seen in PCOS/PMOS (56). AMH has also been implicated in GnRH signaling pathways, with multiple studies demonstrating the presence of AMH receptors on GnRH neurons, suggesting this signaling is upregulated in the setting of higher AMH levels in PCOS/PMOS (57, 58). The increased firing of GnRH neurons is thought to then lead to the elevated LH/FSH ratio, contributing to ovarian HA. The resulting HA decreases the likelihood of recruiting a dominant follicle, resulting in anovulation and PCOM. The involvement of these neuroendocrine pathways in the pathophysiology of PCOS/PMOS presents possible future therapeutic targets.

## Clinical management of PCOS/PMOS

**Figure 2:**
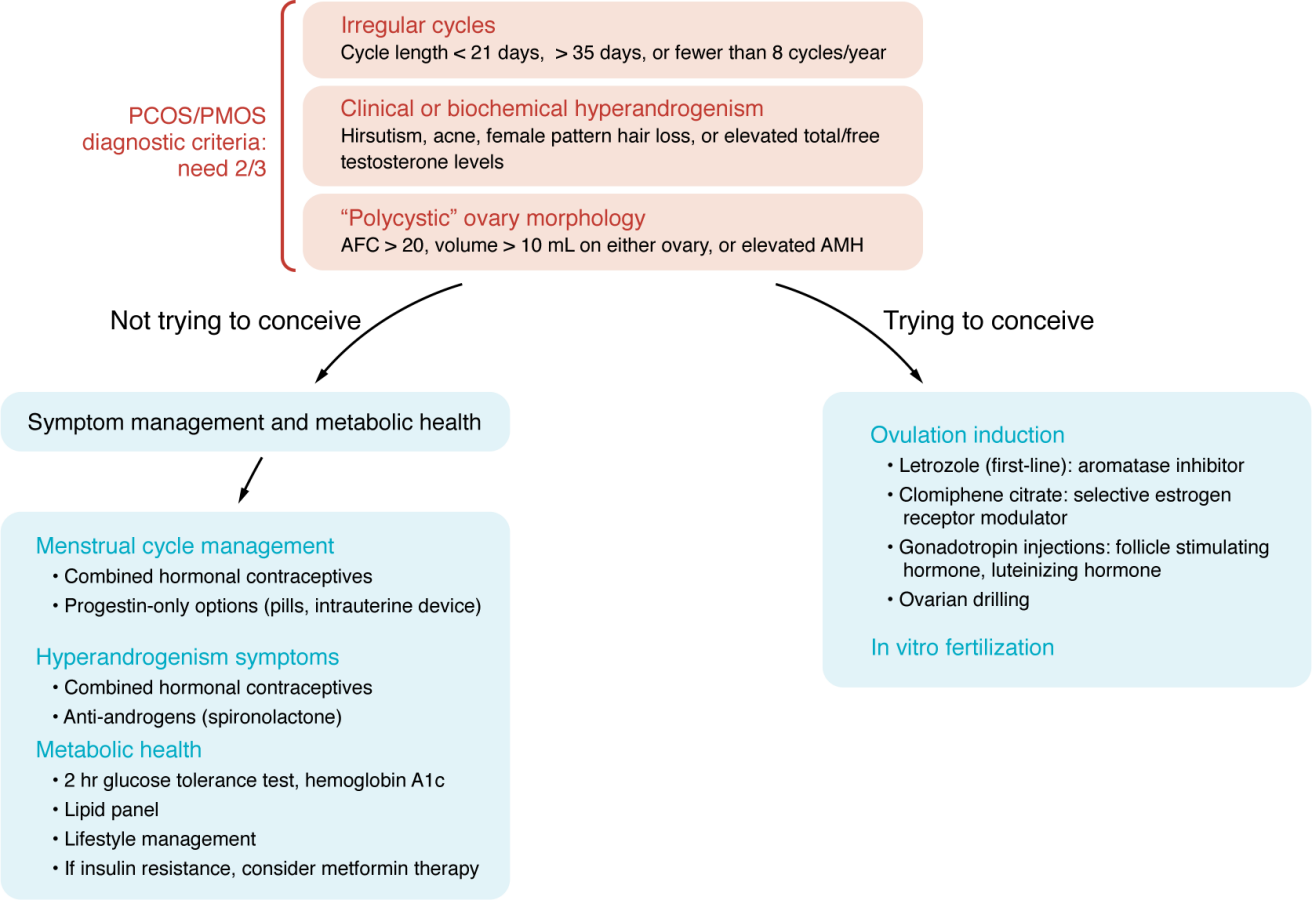
Individualized treatment for PCOS/PMOS. Flow chart describing therapeutic decision-making for individuals with PCOS/PMOS depending on their treatment goals.

The presentation of PCOS/PMOS is extremely heterogeneous; therefore, treatment must be individualized (Figure 2). Although no single medication treats all aspects of PCOS/PMOS, there are several approved therapies that target specific features of the condition, including androgen excess, insulin resistance, and infertility (28). Combined hormonal contraceptives (CHC), which decrease levels of circulating androgens, are a first-line therapeutic agent for both hirsutism and acne (3). When CHC are contraindicated, other antiandrogen options include spironolactone, flutamide, or finasteride, which work at the level of the androgen receptor or androgen production (3). Spironolactone is an aldosterone receptor antagonist that has antiandrogenic effects through inhibition of dihydrotestosterone (DHT) binding. Flutamide is a pure antiandrogen that acts as a competitive antagonist at the level of the androgen receptor. Finasteride is a competitive inhibitor of 5-α reductase, the enzyme that converts testosterone into DHT, leading to lower concentrations of DHT. These medications are teratogenic and should be taken concurrently with contraception. A variety of treatments including pharmaceutical-grade cosmetics, laser treatments, and other methods targeting hair removal or growth can also help address the dermatologic manifestations of HA in PCOS/PMOS (3, 59, 60). Anovulation and resulting abnormal uterine bleeding can also be managed with CHC (61). Ensuring withdrawal bleeding a minimum of every 3 months is important for endometrial protection from hyperplasia and malignancy in individuals with anovulation (62). Weight loss of 5% body weight has also demonstrated improvements in bleeding patterns and restoration of ovulation, particularly in adolescents with PCOS/PMOS (63, 64). Beyond these approaches, there is limited preclinical research on new therapeutic targets including phosphodiesterase-4 inhibitors and interventions modulating the gut microbiome, both of which are thought to target insulin resistance and chronic inflammation (65).

### Metabolic considerations.

A diagnosis of PCOS/PMOS is associated with metabolic consequences and long-term sequelae; therefore, individuals with PCOS/PMOS should have more frequent cardiometabolic risk assessments than their age-matched counterparts (66). As described in the pathophysiology section, PCOS/PMOS is characterized by HA and insulin resistance, which act in concert to promote metabolic dysfunction. HA impairs adipocyte function, encourages visceral fat accumulation, and contributes to insulin resistance, which in turn increases risk of diabetes and heart disease (67). Current guidelines recommend obtaining a lipid profile, a blood pressure measurement, and glucose tolerance testing upon diagnosis of PCOS/PMOS (3). The most accurate method to test glycemic status in the setting of PCOS/PMOS is with a 75 g glucose tolerance test, while measurement of fasting glucose or hemoglobin A1c should be reserved for cases in which the glucose tolerance test is contraindicated (3). After initial screening, blood pressure monitoring should be done at least annually and glycemic testing performed every 1–2 years with the 75 g tolerance test (3). A diagnosis of PCOS/PMOS should be considered an independent risk factor when calculating cardiovascular risk or risk of diabetes (3).

Lifestyle modifications including diet and exercise should be emphasized for all those with PCOS/PMOS and have been shown to improve body fat composition and insulin resistance (68). When weight loss is not achieved with lifestyle changes, there is evidence that bariatric surgery can improve metabolic status of individuals with PCOS/PMOS (47). Metformin should be used for those with insulin resistance, and those diagnosed with diabetes should be managed according to standardized guidelines (66). Metformin is an insulin-sensitizing agent that lowers blood glucose through inhibition of hepatic gluconeogenesis and decreases intestinal glucose absorption. It also increases uptake of glucose in the muscle. Recent data demonstrate ethnic differences in PCOS/PMOS-related metabolic dysfunction, with Black, Hispanic, and Asian women with PCOS/PMOS being at higher risk of insulin resistance (69). On a more global scale, differences in the prevalence of metabolic syndrome and clustering of its components can be seen amid different races and ethnicities, which may reflect contributions of racial and environmental factors (70). These variations highlight the need for comprehensive metabolic screening to identify targets for CVD risk reduction in individuals with PCOS/PMOS.

### Reproductive considerations.

Subfertility in individuals with PCOS/PMOS is thought to arise primarily from oligo-ovulation or anovulation (71), with individuals with HA having lower pregnancy rates (72); therefore, ovulation induction is a mainstay in management. Clomiphene citrate was previously considered the first-line therapy for ovulation induction for those with PCOS/PMOS (62). Clomiphene citrate induces ovulation by acting as a selective estrogen receptor modulator that blocks estrogen receptors in the hypothalamus, reducing negative feedback and increasing release of FSH. FSH then stimulates ovarian follicular development. However, more recent data demonstrate that, for individuals with PCOS/PMOS, ovulation induction with letrozole is associated with higher birth rates compared with clomiphene citrate (27.5% vs. 19.1%) (73, 74). Letrozole is an aromatase inhibitor that creates a peripheral low-estrogen environment that allows for release of FSH. For those unable to achieve pregnancy with ovulation induction, ovarian stimulation with gonadotropins is recommended (75). For those who require in vitro fertilization, there is a higher risk of developing ovarian hyperstimulation syndrome (76). As mentioned previously, even modest weight loss of 5% has been demonstrated to help restore ovulation and therefore potentially improve fertility (63). Metformin can improve ovulatory function; however, it is inferior to other ovulation induction agents and should not be used as a first-line agent for infertility (77, 78). In addition, glucagon-like peptide-1 (GLP-1) receptor agonist therapies, which mimic endogenous GLP-1, increase insulin secretion, and delay gastric emptying, are thought to improve reproductive function (79). A recent study found that a GLP-1/estrogen (GLP-1/E) multiagonist, activating the GLP-1 and estrogen receptors, was especially effective at improving ovulatory function in a preclinical model, offering a possible future therapeutic avenue (80). Importantly, the data on the safety of GLP-1 receptor agonist use in pregnancy are limited, and current guidelines recommend discontinuation when trying to conceive or upon confirmation of pregnancy (81). A recent large retrospective cohort study found increased maternal weight gain and risk of preterm delivery, gestational diabetes, and hypertensive disorders of pregnancy for individuals who used GLP-1 medications shortly before or in early pregnancy (82).

Recent data suggest novel associations between an improved metabolic state and reproductive outcomes in animal models and clinical studies. Caloric restriction in a PCOS/PMOS mouse model prevented the transgenerational inheritance of PCOS/PMOS traits through oocyte-mediated DNA methylation reprogramming (83). Specific diets, including a low-protein, medium-carbohydrate, and medium-fat diet, improved OD but did not improve metabolic dysfunction in a preclinical study using a PCOS/PMOS mouse model (84). A multicenter randomized controlled trial found bariatric surgery to be more effective than medical or behavioral therapy to induce spontaneous ovulation for individuals with PCOS/PMOS and severe obesity (BMI > 35 kg/m²), improving prospects for spontaneous fertility (85). A study on individuals with PCOS/PMOS taking metformin demonstrated partial restoration of endometrial health, which can influence implantation and pregnancy success (86).

Even after achieving pregnancy, either unassisted or through ovulation induction, risk of pregnancy complications remains elevated in women with PCOS/PMOS. There are increased rates of gestational diabetes, hypertensive disorders of pregnancy, and worsened birth outcomes, including increased odds of preterm birth, fetal growth restriction, and low birth weight (87, 88). In pregnant women with PCOS/PMOS who have HA, prenatal androgen exposure may have transgenerational implications on the offspring. Daughters of women with PCOS/PMOS have a longer anogenital distance and increased sebum production (89, 90). These daughters are also five times more likely to be diagnosed with PCOS/PMOS (36). In mice, females with PCOS-like traits induced by DHT injections go on to produce female F__1__–F__3__ offspring with PCOS-like reproductive and metabolic phenotypes. In that study, the authors identified that expression of four candidate genes may be predictive of PCOS/PMOS in future generations: *TIAL1*, *FABP5*, *RNF141*, and *INIP* (36). PCOS/PMOS in pregnancy also has potential health implications for male offspring. One study using a PCOS/PMOS mouse model demonstrated that male offspring are at an increased risk for obesity and dyslipidemia, and these traits may be transmitted to subsequent generations via small noncoding RNAs (91). Sequencing sperm from F__1__–F__3__ male mice born to androgenized PCOS-like mice demonstrated distinct differentially expressed small noncoding RNAs across generations, suggesting a risk of transmission of reproductive and metabolic dysfunction through the male germline (91). In humans, a register-based cohort, clinical case-control study demonstrated that the sons of women with PCOS/PMOS are more often obese and dyslipidemic (91). The mechanisms underlying the transgenerational effects of PCOS/PMOS require further study in preclinical models and human cohorts.

## Long-term health sequelae of PCOS/PMOS

### Cardiovascular risk.

Individuals with PCOS/PMOS are at an elevated risk for obesity, insulin resistance and diabetes, hypertension, and dyslipidemia, which places them at greater risk for CVD (92). Despite this association, a definitive causal link between PCOS/PMOS and CVD has not been established (93). Due to the variability in diagnosing PCOS/PMOS and definitions of CVD across studies, it is difficult to determine to what extent PCOS/PMOS itself or its comorbidities modulate the risk for CVD. Prospective longitudinal studies with long follow-up in a well-defined PCOS/PMOS population, potentially with genetic markers, will better elucidate this relationship. Nonetheless, given the apparent risk, CVD risk factor screening in the form of early glucose and lipid screening is recommended in all individuals diagnosed with PCOS/PMOS, as early intervention, such as lifestyle management and pharmacotherapy, may have long-term health benefits.

Even in young women with PCOS/PMOS, there is evidence of increased subclinical CVD compared with those without PCOS/PMOS (92, 94), with abnormal physiologic alterations occurring at the level of the vascular endothelium. Endothelial dysfunction can be measured in a variety of ways, including flow-mediated dilation, arterial stiffness, coronary artery calcium scores, carotid intima-media thickness, and more novel markers such as visceral and epicardial fat (92). These measures of subclinical CVD have yet to be adopted into standard practice to follow individuals with PCOS/PMOS long term. Future longitudinal studies will allow for a better understanding of the role of these subclinical CVD markers in major cardiovascular events in PCOS/PMOS.

### Insulin resistance.

Despite the high prevalence of insulin resistance among individuals with PCOS/PMOS, there are challenges in measuring and estimating insulin resistance in the clinical setting. The euglycemic hyperinsulinemic clamp is the gold standard method of measuring insulin resistance. However, it is a labor-intensive, technically demanding test that is not practical for use in the clinical setting (95, 96). Several surrogate markers including insulin and glucose measured during an oral glucose tolerance test (OGTT) and a Homeostatic Model Assessment of Insulin Resistance (HOMA-IR) score have been suggested for use in PCOS/PMOS (95).

### Mental health.

There is an increased risk of depression, anxiety, bipolar disorder, and obsessive-compulsive disorder (OCD) among individuals diagnosed with PCOS/PMOS (2). The association is particularly strong between PCOS/PMOS, depression, and anxiety, with those with PCOS/PMOS having nearly three times the odds of depressive symptoms and four times the odds of anxiety symptoms (2). While fewer in number, several studies support the increased risk for both bipolar disorder and OCD (97). This supports the need for clinician education, as well as for implementing early screening strategies and intervention for individuals with PCOS/PMOS (2).

### Risk of cancer.

There is an increased risk for endometrial cancer in women of all ages with PCOS/PMOS. However, due to the confounding factors of anovulation, obesity, and diabetes mellitus for PCOS/PMOS and endometrial cancer, it is unclear if the risk of endometrial cancer is due to PCOS/PMOS itself or its known comorbidities (98, 99). It has also been hypothesized that there is a common genetic variant between PCOS/PMOS and endometrial cancer, though this genetic correlation was decreased when adjusting for predicted BMI, indicating that this genetic risk may be mediated by obesity (100).

## Future directions

### Role of biomarkers.

Due to the controversial and varied diagnostic criteria used to define PCOS/PMOS, approximately 70% of PCOS/PMOS cases may be undetected (101). Detection of early-stage PCOS/PMOS with biomarkers may lead to a more timely and potentially more accurate identification of individuals with disease to allow for early intervention and prevention of disease comorbidities.

### Role of genetics and epigenetics.

Future studies focusing on the contribution of genetics in PCOS/PMOS are needed. As with other complex traits, modern genetic analyses have shed light on the importance of genetic variation in the pathogenesis of PCOS/PMOS (23). Genetic subtyping has identified different reproductive and metabolic phenotypes of PCOS/PMOS. In addition, epigenetic changes through environmental exposures in utero could influence PCOS/PMOS phenotyping across generations. Animal data support the role of intrauterine androgen programming in PCOS/PMOS development in offspring (36, 102). In concert with genetic susceptibility, this androgen excess could impact the development of PCOS/PMOS in an epigenetic fashion. Understanding the contributions of both genetics and epigenetics in PCOS/PMOS would change the landscape of targeted therapies and prevention.

### Role of bioinformatics and machine learning.

The potential of artificial intelligence–based technologies, such as machine learning algorithms, is under investigation in the detection of complex medical diseases, such as heart disease (103). The same methods can be applied to improving detection of PCOS/PMOS through identification of patterns in medical data, such as hormone levels (104). Improved accuracy of diagnosis of PCOS/PMOS through machine learning could provide earlier diagnosis and more precise intervention. Through creation of predictive models using machine learning methods and hormone values (FSH, LH, estradiol, and SHBG), Zad et al. were able to predict PCOS/PMOS prior to clinical diagnosis in an out-of-sample test of patients with high accuracy (area under the curve as high as 85%) (105). This technology could also be used to monitor individuals over time to determine which interventions are most effective.

### Treating the reproductive and hormonal manifestations of PCOS/PMOS.

Currently, treatment strategies for PCOS/PMOS are primarily symptomatic. Selection of pharmacologic therapy is targeted at the individual characteristics of the patient, as described above. Limitations of PCOS/PMOS animal models and epidemiologic studies have made it difficult to develop targeted drug therapies (106). Due to the complex mechanisms of PCOS/PMOS and variable presentation of the disease from person to person, optimal treatment would involve a precision medicine approach.

As we understand more about the genetics and epigenetics of PCOS/PMOS, there is the prospect of targeted therapy. In polygenic diseases, such as PCOS/PMOS, abnormalities can converge on key shared biologic pathways, which are potential targets for treatment. For example, kisspeptin is an upstream regulator of GnRH signaling, which plays a critical role in steroid hormone feedback. Treatment based on the kisspeptin receptor agonist MVT-602 has demonstrated effectiveness in inducing oocyte maturation and ovulation (107, 108). There are also studies evaluating the relationship among fat mass, the fat mass and obesity associated gene (*FTO*), and PCOS/PMOS clusters related to obesity and fat distribution. CRISPR-based editing of *FTO* rs1421085 in primary adipocytes from a patient with PCOS/PMOS resulted in a 7-fold increase in fat cell thermogenesis, indicating this allele could be a target for the obese PCOS/PMOS subtype (109).

Neurokinin 3 (NK3) receptor antagonism at the level of kisspeptin, neurokinin B, and dynorphin A neurons has been implicated in the decrease of GnRH pulse frequency leading to decreased LH secretion, lower LH/FSH ratio, and suppressed follicular growth (56). Studies have explored the role of NK3 receptor antagonists, such as fezolinetant, in restoring GnRH pulse frequency. In a clinical study, fezolinetant had a sustained effect to suppress HA and reduce the LH/FSH ratio in women with PCOS/PMOS (110).

The artemisinin analog artemether, a known antimalarial agent, has been demonstrated to alleviate symptoms associated with PCOS/PMOS in rodent models and humans via direct binding with lon peptidase 1 (LONP1), promoting degradation of the cytochrome P450 family 11 subfamily A member 1 (CYP11A1), and inhibiting ovarian androgen synthesis (111). Treatment with artemether led to improvement of HA, irregular estrous cycles, PCOM, and subfertility in PCOS-like rodent models. In a small pilot clinical trial in human participants with PCOS/PMOS, dihydroartemisinin led to decreased HA, lower AMH levels, reduced PCOM, and normalized menstrual cycles (111). This is an example of drug repurposing that could lead to new treatments for PCOS/PMOS.

### Treating the metabolic manifestations of PCOS/PMOS.

GWAS data can also be used to guide drug repurposing, identifying new indications for existing medications (112). For example, studies have identified shared risk loci among obesity, diabetes, and PCOS/PMOS, such as *GIPR* and *GLP-1R* (113). These incretin receptors are targets for medications approved for the treatment of obesity and diabetes, and given the shared underlying genetics between obesity and PCOS/PMOS, the drugs may prove effective for PCOS/PMOS as well (113, 114). Indeed, GLP-1 receptor agonists have also been demonstrated to treat metabolic syndrome and decrease BMI, waist circumference, and insulin resistance for individuals with PCOS/PMOS, especially in the setting of obesity (115, 116). Animal studies have suggested that GLP-1 polyagonists, which simultaneously activate multiple metabolic hormone receptors, such as GLP-1 and estrogen receptors, have shown even more promise for PCOS/PMOS (80, 117). In a 2024 study, Sánchez-Garrido and colleagues compared the use of metformin with GLP-1–based multiagonists, GLP-1/E, GLP-1/gastric inhibitory peptide (GLP-1/GIP), and GLP-1/GIP/glucagon, in two mouse models of PCOS/PMOS with variable penetrance of metabolic and reproductive traits (80). They observed superior efficacy of GLP-1/E versus other multiagonists and metformin on metabolic complications of PCOS/PMOS, as well as improved ovarian cyclicity in the ovulatory model.

Another potential target is aldo-keto reductase family 1 member C3 (AKR1C3). AKR1C3 is an enzyme that leads to androgen production and activation of the androgen receptor and is induced by insulin in adipocytes from patients with PCOS/PMOS. Studies of AKR1C3 in differentiated PCOS/PMOS adipocytes have elucidated two roles in PCOS/PMOS: 1) to catalyze potent androgen formation in adipocytes leading to HA and 2) to induce fatty acid synthase for de novo lipogenesis by stabilizing androgen receptors in the absence of androgens. An in vivo phenotyping study in women with PCOS/PMOS demonstrated that insulin stimulated adipose AKR1C3 expression and activity, whereas exposure to androgens increased de novo lipid synthesis in adipocytes (90). Therefore, pharmacologic inhibition of AKR1C3 may be a potential approach to decrease lipid accumulation and insulin resistance in PCOS/PMOS.

### Microbiome considerations.

The association between the gut microbiota and endocrine function is an area of interest that may influence future treatment strategies for PCOS/PMOS. Gut microbiota–produced metabolites can affect the secretion of hormones (118). Altered gut microbiota composition, or dysbiosis, has been associated with the pathogenesis of many diseases and can lead to metabolic and endocrine dysfunction. Recent studies demonstrate that individuals with PCOS/PMOS have alterations to the gut microbiota compared with controls (119). *Bifidobacterium* and Enterobacteriaceae were found to be more prevalent in PCOS/PMOS, whereas *Prevotella* was reduced (120). Functional analyses of the gut microbiota of individuals with PCOS/PMOS demonstrated alterations in pathways related to short-chain fatty acid production (121). Gut microbiota composition influences insulin sensitivity and secretion, as well as obesity and inflammatory response (122), all thought to be altered in PCOS/PMOS. Targetiereng dysbiosis and restoration of the gut microbiota could be a future individualized treatment strategy for PCOS/PMOS (122). Indeed, early preclinical data suggest interventions such as prebiotics, probiotics, and fecal microbiota transplantation can modulate inflammatory and oxidative pathways that contribute to the metabolic dysfunction in PCOS/PMOS (65).

### PCOS/PMOS models.

Finally, new models of PCOS/PMOS can facilitate new therapeutic discoveries. Through the use of a microfluidic platform that can house up to eight organ models to mimic the body’s microenvironment (the LATTICE platform), PCOS/PMOS can be studied in three dimensions in vitro, providing more robust and rich information compared with traditional two-dimensional cell culture systems (123). When exposed to androgens and gonadotropins to mimic PCOS/PMOS endocrinology, this platform enables the study of changing hormone profiles seen in PCOS/PMOS. Additionally, this platform allows for automated, high-throughput drug testing that could fast-track drug discovery for PCOS/PMOS treatment. These organs-on-a-chip can be applied to PCOS/PMOS to open the door for individualized treatment and personalized medicine for this heterogeneous disease (124).

## Conclusion

While PCOS/PMOS is the most common endocrinopathy in reproductive-aged women, the pathophysiology of the disorder remains largely unclear. PCOS/PMOS has far-reaching health implications from menstrual irregularities, HA symptoms, and infertility to elevated risk for long-term health issues, including cancer, CVD, metabolic syndrome, and mental health disorders. PCOS/PMOS is difficult to study due to the heterogeneity in presentation and variable phenotypes. Despite these challenges, studies in the genetics and epigenetics arena, as well as exploring the gut microbiota, in PCOS/PMOS are underway. The results will be invaluable in defining PCOS/PMOS more clearly and will allow for personalized approaches to prevention and treatment.

## Conflict of interest

The authors declare that no conflict of interest exists.

## Figures and Tables

**Table 2:**
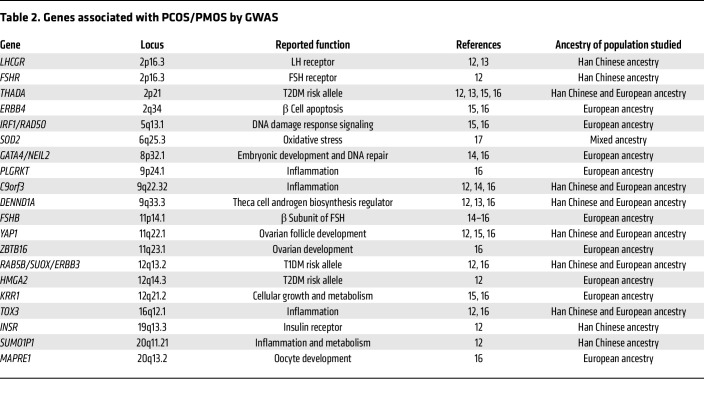
Genes associated with PCOS/PMOS by GWAS

**Table 1:**
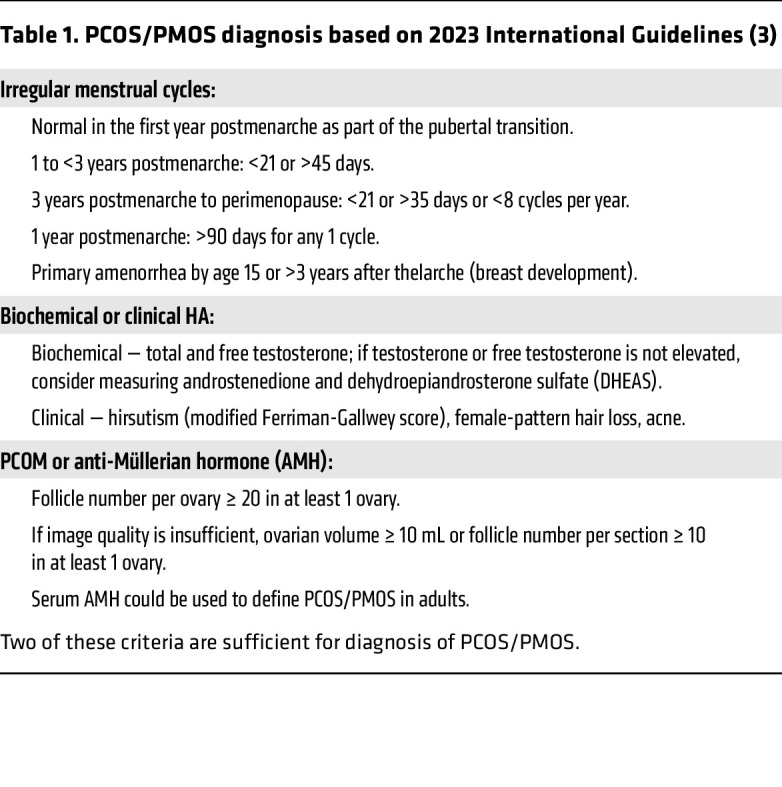
PCOS/PMOS diagnosis based on 2023 International Guidelines (3)

## Version 1 06/15/2026

Electronic publication
